# Bibliometric analysis of worldwide scientific literature in mobile - health: 2006–2016

**DOI:** 10.1186/s12911-017-0476-7

**Published:** 2017-05-30

**Authors:** Waleed M. Sweileh, Samah W. Al-Jabi, Adham S. AbuTaha, Sa’ed H. Zyoud, Fathi M. A. Anayah, Ansam F. Sawalha

**Affiliations:** 1 Department of Physiology, Pharmacology and Toxicology, College of Medicine and Health Sciences, An-Najah National University, Nablus, Palestine; 2 Department of Clinical and Community Pharmacy, College of Medicine and Health Sciences, An-Najah National University, Nablus, Palestine; 3 College of Engineering and Technology, Palestine Technical University-Kadoorie (PTUK), Technical University - Kadoorie, P.O. Box 7, Yafa Street, Tulkarm, Palestine

**Keywords:** Mobile Health, Bibliometric, VOSviewer

## Abstract

**Background:**

The advancement of mobile technology had positively influenced healthcare services. An emerging subfield of mobile technology is mobile health (m-Health) in which mobile applications are used for health purposes. The aim of this study was to analyze and assess literature published in the field of m-Health.

**Methods:**

SciVerse Scopus was used to retrieve literature in m-Health. The study period was set from 2006 to 2016. ArcGIS 10.1 was used to present geographical distribution of publications while VOSviewer was used for data visualization. Growth of publications, citation analysis, and research productivity were presented using standard bibliometric indicators.

**Results:**

During the study period, a total of 5465 documents were published, giving an average of 496.8 documents per year. The *h-*index of retrieved documents was 81. Core keywords used in literature pertaining to m-Health included diabetes mellitus, adherence, and obesity among others. Relative growth rate and doubling time of retrieved literature were stable from 2009 to 2015 indicating exponential growth of literature in this field. A total of 4638 (84.9%) documents were multi-authored with a mean collaboration index of 4.1 authors per article. The United States of America ranked first in productivity with 1926 (35.2%) published documents. India ranked sixth with 183 (3.3%) documents while China ranked seventh with 155(2.8%) documents. *VA Medical Center* was the most prolific organization/institution while *Journal of Medical Internet Research* was the preferred journal for publications in the field of m-Health. Top cited articles in the field of m-Health included the use of mobile technology in improving adherence in HIV patients, weight loss, and improving glycemic control in diabetic patients.

**Conclusion:**

The size of literature in m-Health showed a noticeable increase in the past decade. Given the large volume of citations received in this field, it is expected that applications of m-Health will be seen into various health aspects and health services. Research in m-Health needs to be encouraged, particularly in the fight against AIDS, poor medication adherence, glycemic control in Africa and other low income world regions where technology can improve health services and decrease disease burden.

**Electronic supplementary material:**

The online version of this article (doi:10.1186/s12911-017-0476-7) contains supplementary material, which is available to authorized users.

## Background

Mobile health (mHealth or m-Health) was defined more than a decade ago as the use of mobile communication devices in health services or as an emerging application of mobile technologies for healthcare systems [1–3]. The world health organization (WHO) defined m-Health as *“medical and public health practice supported by mobile devices, such as mobile phones, patient monitoring devices, personal digital assistants (PDAs), and other wireless devices”* [4]. Mobile health is a relatively recent field that emerged as a branch or component of health which involves the use of wireless technology or electronic processes in health services [5]. Many published reports indicated that m-health had a positive impact on individual and national health services [6]. Mobile health had been applied in a wide range of health services including promoting medication adherence, prevention of behaviors associated with certain diseases, psychological support for patients with chronic diseases, weight loss, smoking cessation and many others [7–18].

Bibliometrics and scientometrics are defined as mathematical/statistical methods used to assess the quality and quantity of published scientific literature and to study research trends, citation analysis, authorship, impact of publications, journal analysis, as well as national and international contribution in a particular field. Bibliometrics and scientometrics had been applied to many scientific fields including mobile technology and telecommunication [19–23]. However, up until this date, no bibliometric analysis of scientific literature in m-Health had been carried out and published. Therefore, the aim of this study was to analyze published literature in m-Health. In specific, growth of publications, country contribution, international collaboration, citation analysis, keyword occurrences, authorship analysis, most productive institutions, and most productive journals in the field of m-Health were investigated.

## Methods

### Bibliographic database

In this study, data pertaining to m-Health were retrieved from Scopus, a bibliographic database that covers nearly 22,000 titles in the scientific, technical, medical, and social sciences. The choice of Scopus was made based on the idea that it is the largest database when compared to Pubmed or Web of Science [24]. Furthermore, several analytical functions can be performed using Scopus (Additional file 1). One such function was “source type” which allowed us to refine retrieved literature based on type of data source whether the source was journal publications or books or book chapters or conference proceedings or trade publications. In this study, we limited our analysis to documents published in journals but not in books or conference proceedings (Additional file 1). Conference proceedings included abstracts which might have been published twice as a conference abstract and as a full journal article and therefore might create false positive results. Once the source type was limited to journal publications, the type of these publications can be explored using the function “document type” (Additional file 1). In document type, errata documents, corrections of an already published articles, were excluded from the analysis since they do not represent true publications. The conference papers that appeared under document type were different from those that appeared under source type. In document type, conference papers refer to papers presented in conferences and were published as full journal articles and therefore could not be published twice. This approach of limiting source data to journal publications will minimize false positive, particularly for those which originated from conference proceedings.

### Search strategy and validity

Our search strategy was based on two approaches that were combined at a later step. The first approach included search for journal publications in which the term (“mobile health”) appeared in the title or abstract or author keywords. However, since not all publications in mobile health could be retrieved this way, we had to use a second approach in which we searched for all possible documents that included in their titles words related to mobile technology such as smartphone or texting or cellular phone or mobile application plus a word in any field of health. In each approach, the time was set from 2006 to 2016 and source type was limited to journal publications. The number of documents retrieved using each approach was shown in Additional file 1. The first approach yielded a total of 2452 (Additional file 1) while the second approach yielded a total of 3263 (Additional file 1) and when combined, they yielded a total of 5465 journal publications. The overlap between the two approaches was 250 documents. One might ask why search for the term “mobile health” did not yield all potential documents and the answer for that was the tendency of some researchers to use terms such as smartphones or text message in addition to the disease or ailment being investigated instead of using the recently defined term “m-Health”. Furthermore, some researchers might use broader terms such as telemedicine. Therefore, it was not surprising that the term “mobile health” did not retrieve all needed documents and the use of a second detailed approach was needed. The overall search strategy was shown in Additional file 1. In the second approach, the health related terms were obtained from several systematic reviews and review articles in m-Health to ensure conclusiveness of the search strategy [25–33]. For validity of the search strategy, two co-authors manually reviewed 110 articles chosen as top 10 cited articles for each year in the study period. For all manually reviewed articles, no false positive results were obtained. Furthermore, the preferred journal names in which publications appeared indicated that the retrieved articles were within the correct scope.

### Data analysis

Retrieved data were analyzed for document type, annual growth, citation analysis, authorship, country productivity, and for articles with the highest number of citations. Document types were presented as frequency and percentage. For annual growth, we presented data as number of retrieved documents in each year. Furthermore, the annual growth rate (AGR), defined as the percentage change in the number of publications over a period of 1 year was calculated based on the following equation: AGR = [(Ending Value - Beginning Value)/Beginning Value] * 100.

The growth analysis was also presented as relative growth rate (RGR) which was defined as the increase in number of publications per unit of time. The RGR was calculated based on the following equation: *RGR = [log*_*e*_ *W*_*2*_ *- log*_*e*_ *W*_*1*_ *]/(T2 - T1)* [19, 34, 35] where log_e_ W_1_: log of initial number of articles; log_e_ W_2_: log of final number of articles after a specific period of interval; and T2-T1: the unit difference between the initial time and the final time. The RGR can be presented in a different format called doubling time (DT), defined as the period of time required for the number of publications to double in number in 1 year and was calculated based on the following equation: *DT = 0.693/RGR* [19, 34, 35]. Finally, the citation analysis was presented as total citations, mean and median (Q1 – Q3) citations. The quality of publications was measured using Hirsch index (h-index) and was obtained directly from Scopus.

### Collaboration and authorship analysis

Author details were exported from Scopus to Microsoft Excel where authorship analysis was carried out. Analysis in Microsoft Excel included number of single-authored articles and number of multi-authored (joint) articles. Analysis of overall collaboration in the field of m-Health was calculated using the following equation: Degree of collaboration = *C = N*_*m*_ */N*_*m*_ *+ N*_*s*_ [19, 34, 35] where N_m_ = number of multi-authored papers and N_s_ = number of single-authored papers. The collaboration index (CI) was calculated based on the number of multi-authored (joint) papers and the number of authors of these multi-authored (joint) papers. The equation used to calculate the collaboration index was as follows: CI = number of authors in joint articles/number of joint papers [19, 34, 35]. Authorship analysis included analysis of average number of authors per paper which was calculated as: (AAPP) = total number of authors/total number of papers.

### Visualization and mapping

Retrieved data were visualized using VOSviewer program, a software tool used for visualization of bibliometric networks. In visualization techniques, maps could be presented as a density map or as a network visualization in which color, circle size, font size, and thickness of connecting lines were used to present certain parameters. For example, units with similar color indicated that these units belonged to one cluster or group of close units that could be countries or authors. The strength of collaboration between countries was measured by the thickness of connecting lines which was numerically represented as relative link strength. Higher relative link strength suggested stronger collaboration. The larger circle size or font size indicated greater productivity or citations [36]. For geographical distribution of publications ArcGIS 10.1 (ArcMap 10.1), a geographic information system (GIS), was used. To create the geographical distribution map, retrieved data were exported to excel and then were transferred to ArcGIS 10.1 program to create the GIS map.

### Statistical analysis and ethics

Descriptive statistics for categorical variables such as frequencies and percentages were presented. For continuous data, mean, median, and range were represented. For median, the interquartile range (Q1-Q3) was presented. No statistical testing was carried out. For data analysis and presentation, Microsoft Excel and Statistical Package for Social Sciences (SPSS 20) were used. Graphics was made using SPSS 20. This study included no human subjects and is based on electronic data and therefore it was exempted from ethical approval.

## Results

### Description of retrieved literature

**Fig. 1:**
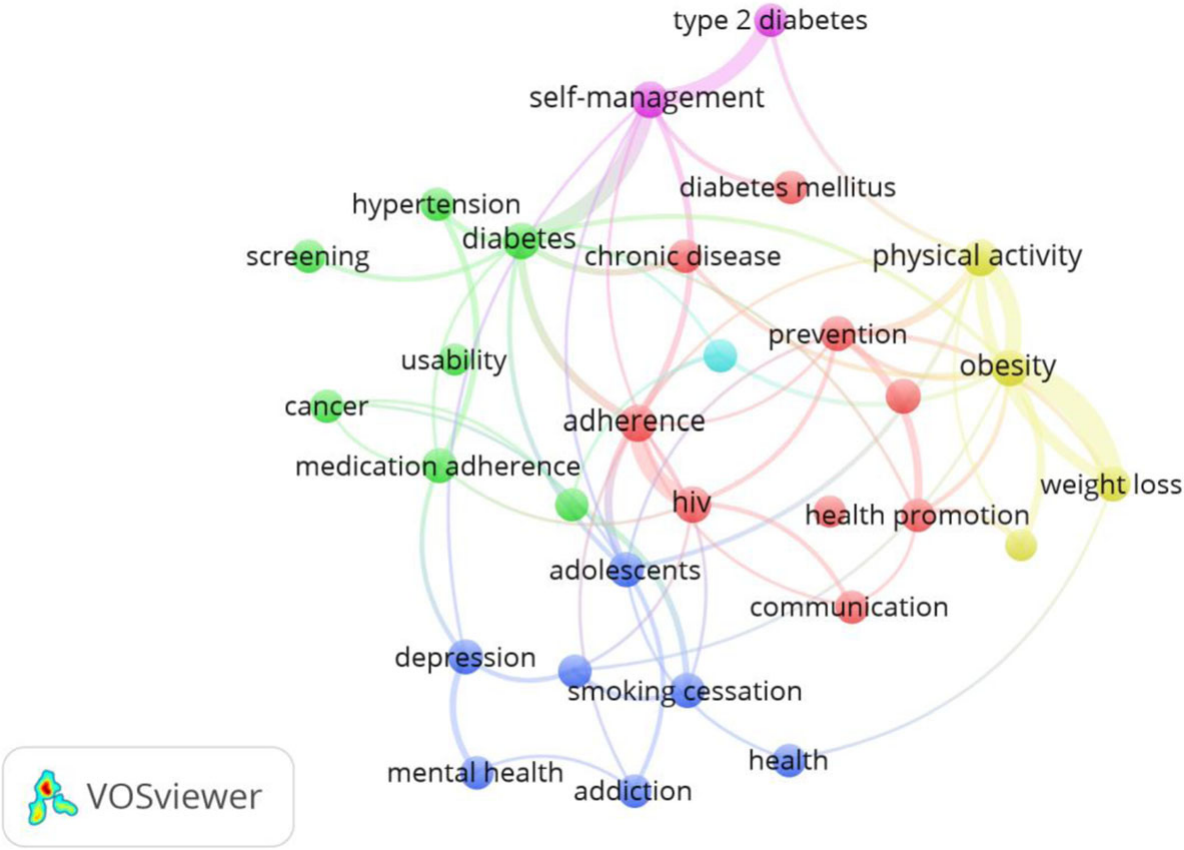
Network visualization map of author keywords occurrences (i.e., keywords listed by the author). Keywords with minimum occurrences of 25 times were shown in the map. Keywords with the same color were commonly listed together. So, for example, physical activity, obesity, health behavior, and weight loss have similar color suggestive that these keywords have close relation and usually co-occur together

A total of 5465 documents were retrieved; the majority (4194; 76.7%) were research articles. The second most common type of document was review articles which constituted 9.6%. Details about types of retrieved documents were shown in Table 1. The majority of retrieved documents were published in English (5118; 93.7%). Other commonly encountered languages included French (130; 2.4%) and German (72, 1.3%). Retrieved documents received a total of 54937 citations, a mean of 10.8 ± 31 citations per document; median (Q1-Q3) of 2 (0–10), and a range of 449 (0–449). The *h*-index of retrieved documents was 81. Mapping with VOSviewer technique of author keywords with minimum occurrences of 25 showed that keywords such as type 2 diabetes/diabetes mellitus; HIV, medication adherence, obesity, weight loss, physical activity, mental health, hypertension, health promotion, smoking cessation, addiction, depression, and self-management were most encountered author keywords after exclusion of the core keywords related to search query (Fig. 1).

**Table 1:** Types of retrieved documents (2006–2016)

Type of document	Frequency	% *N* = 5465
Article	4194	76.7
Review	523	9.6
Note	199	3.6
Letter	198	3.6
Article in Press	116	2.1
Editorial	91	1.7
Conference Paper	78	1.4
Short Survey	66	1.2
Total	5465	100%

### Growth of publications

The mean number of publications during the study period was 496.8 documents per year. The highest productivity was observed in 2016 with a total of 1095 (20.0%) documents and the lowest productivity was in 2006 with a total productivity of 155 (2.8%). There was an increase in the number of publications during the study period. The total number of citations per year for retrieved documents is shown in Table 2. The number of citations per document was highest for documents published in 2009 (21.2 citations per document) while the lowest was for those published in 2016 (0.8 citations per document) due to short time elapsed since publications. Growth analysis indicated that AGR had a fluctuating pattern during the study period (Table 3). RGR declined from 0.8 in 2007 to 0.3 in 2009 and then remained stable at 0.3 from 2009 to 2015 followed by a decline to 0.2 in 2016. The DT increased from 0.9 in 2007 to 2.3 in 2009 and continued to remain at 2.3 until 2015 followed by an increase in 2016 to 3.5. Stable values of RGR and DT indicated exponential growth of publications.

**Table 2:** Annual number of publications and citation analysis per year (2006 – 2016)

Year	Frequency	% *N* = 5465	TC	Mean ± SD of citations	Median (Q1 – Q3) of citations
2006	155	2.8	2438	15.7 ± 33.8	5 (1–17)
2007	176	3.2	3248	18.5 ± 31.5	11 (1–21.75)
2008	189	3.5	3646	19.3 ± 32.6	6 (1–20.5)
2009	234	4.3	4955	21.2 ± 42.7	6 (1–23)
2010	288	5.3	5994	20.8 ± 45.6	7 (1–24.75)
2011	343	6.3	6886	16.6 ± 39.7	4 (0–17)
2012	505	9.2	8332	20.1 ± 26.7	7 (2–21)
2013	630	11.5	8581	13.6 ± 23.4	6 (2–16)
2014	861	15.8	6179	7.2 ± 11	3 (1–9)
2015	989	18.1	3749	3.8 ± 6.7	2 (0–4.5)
2016	1095	20.0	929	0.8 ± 1.8	0 (0–1)

*TC* Total citations, *SD* Standard deviation, Q1-Q3 Interquartile range

**Table 3:** Annual number of publications, AGR, RGRT, and DT (2006–2016)

Year	Frequency	AGR	Cumulative total publications	Log_e_ *W*	RGR	DT
2006	155	-	155	5.0	-	-
2007	176	13.5	331	5.8	0.8	.9
2008	189	7.4	520	6.3	0.5	1.4
2009	234	23.8	754	6.6	0.3	2.3
2010	288	23.1	1042	6.9	0.3	2.3
2011	343	19.1	1385	7.2	0.3	2.3
2012	505	47.2	1890	7.5	0.3	2.3
2013	630	24.8	2520	7.8	0.3	2.3
2014	861	36.7	3381	8.1	0.3	2.3
2015	989	14.9	4370	8.4	0.3	2.3
2016	1095	10.7	5465	8.6	0.2	3.5

AGR: annual growth rate; RGR: relative growth rate; DT: doubling time

### Authorship pattern, collaboration, and prolific authors

**Fig. 2:**
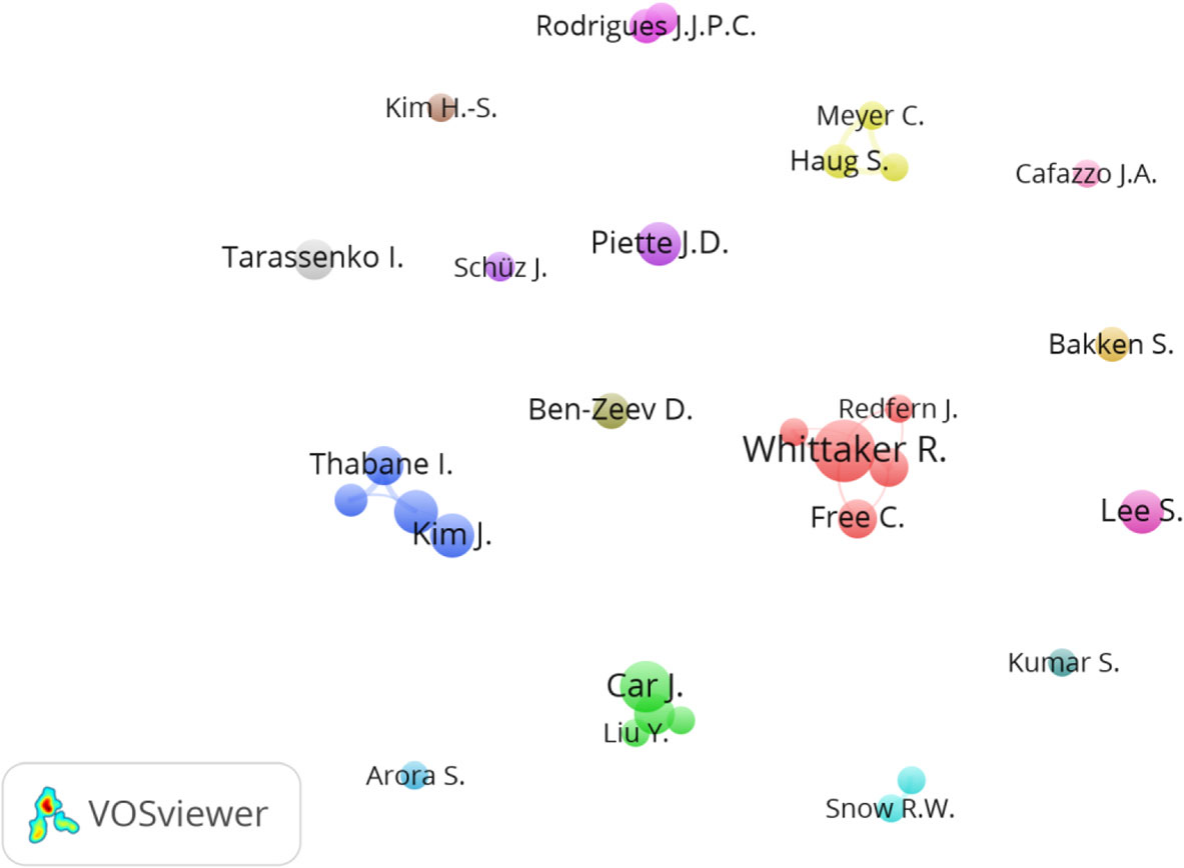
Network visualization map of active authors in m-Health. Authors with a minimum of 10 publications and 100 citations were visualized. The map included 30 authors who met the criteria of being an active author. Some names might not be seen due to overlap of names. Closed circles indicated active authors of close research collaboration

A total number of 23554 researchers participated in publishing retrieved documents giving a mean of 4.0 authors per document. The mean number of authors per document showed an increasing trend with time; from 3.3 in 2006 to 5.1 in 20016 (Table 4). A total of 827 (15.1%) documents were single-authored publications while the remaining documents (4638; 84.9%) were multi-authored publications. Therefore, prevalence of team research or the degree of research collaboration among researchers in m-Health was 84.9%. The collaboration index (CI) for multi-authored documents increased from 3.8 in 2006 to 4.6 in 2016; with a mean of 4.1 authors per document in multi-authored (joint) publications (Table 5). Authors with a minimum productivity of 10 documents and a minimum total citation of 100 were visualized using VOSviewer technique and presented in Fig. 2. The map included 30 circles, each representing one author. Closer circles indicate authors with close research collaboration.

**Table 4:** Average author per documents and author productivity per year (2006–2016)

Year	Frequency	% *N* = 5465	Total number of authors	Average number of authors per document
2006	155	2.8	515	3.3
2007	176	3.2	696	4.0
2008	189	3.5	702	3.7
2009	234	4.3	820	3.5
2010	288	5.3	1092	3.8
2011	343	6.3	1352	3.9
2012	505	9.2	2165	4.3
2013	630	11.5	2739	4.3
2014	861	15.8	3943	4.6
2015	989	18.1	3962	4.0
2016	1095	20.0	5568	5.1
Total	5465	100	23554	4.0

**Table 5:** Collaboration index (CI) among authors in m-Health field (2006–2016)

Year	Frequency	% *N* = 5465	Total number of authors	Number of single authored publications	%	Number of multi- authored publications	%	number of authors in multi-authored publications	CI
2006	155	2.8	515	48	31.0	107	69.0	408	3.8
2007	176	3.2	696	36	20.5	140	79.5	556	4.0
2008	189	3.5	702	43	22.8	146	77.2	556	3.8
2009	234	4.3	820	65	27.8	169	72.2	651	3.9
2010	288	5.3	1092	59	20.5	229	79.5	863	3.8
2011	343	6.3	1352	76	22.2	267	77.8	1085	4.1
2012	505	9.2	2165	87	17.2	418	82.8	1747	4.2
2013	630	11.5	2739	95	15.1	535	84.9	2204	4.1
2014	861	15.8	3943	106	12.3	755	87.7	3188	4.2
2015	989	18.1	3962	117	11.8	872	88.2	3090	3.5
2016	1095	20.0	5568	95	8.7	1000	91.3	4568	4.6
Total	5465	100	23554	827	15.1	4638	84.9	18916	4.1

CI: Collaboration index

### Geographical distribution of publications

**Fig. 3:**
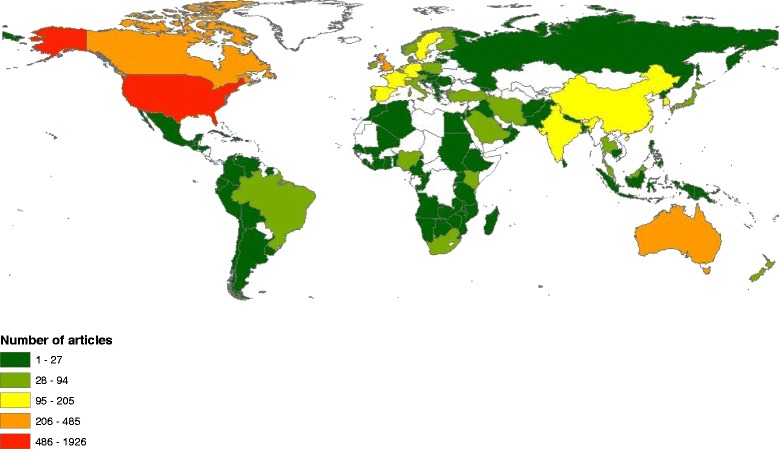
Geographical distribution of publication in m-Health. The map was created using ArcGIS 10.1 software. The map is color coded where world regions with *red* color had the highest productivity while world regions with dark *green* had the lowest productivity. Areas with no color indicates no data available from these areas

Researchers from 120 different countries contributed to the publication of retrieved documents. The distribution of publications on world map using ArcMap 10.1 software was shown in Fig. 3. Countries with a minimum productivity of 50 publications are listed in Table 6. The United States of America (USA) ranked first with a total of 1926 (35.2%) documents followed by the United Kingdom (UK) and Australia.

**Table 6:** Countries with a minimum productivity of 50 documents in the field of mobile health

Rank	Country	Frequency	% *N* = 5465
1	United States	1926	35.2
2	United Kingdom	485	8.9
3	Australia	280	5.1
4	Canada	268	4.9
5	South Korea	205	3.8
6	India	183	3.3
7	Germany	173	3.2
8	China	155	2.8
9	Spain	153	2.8
10	France	149	2.7
11	Netherlands	116	2.1
12	Sweden	113	2.1
13	Switzerland	106	1.9
14	Italy	94	1.7
15	South Africa	80	1.5
16	Japan	72	1.3
16	Taiwan	72	1.3
18	New Zealand	65	1.2
19	Turkey	60	1.1
20	Denmark	55	1.0
21	Iran	54	1.0
22	Norway	50	0.9

**Fig. 4:**
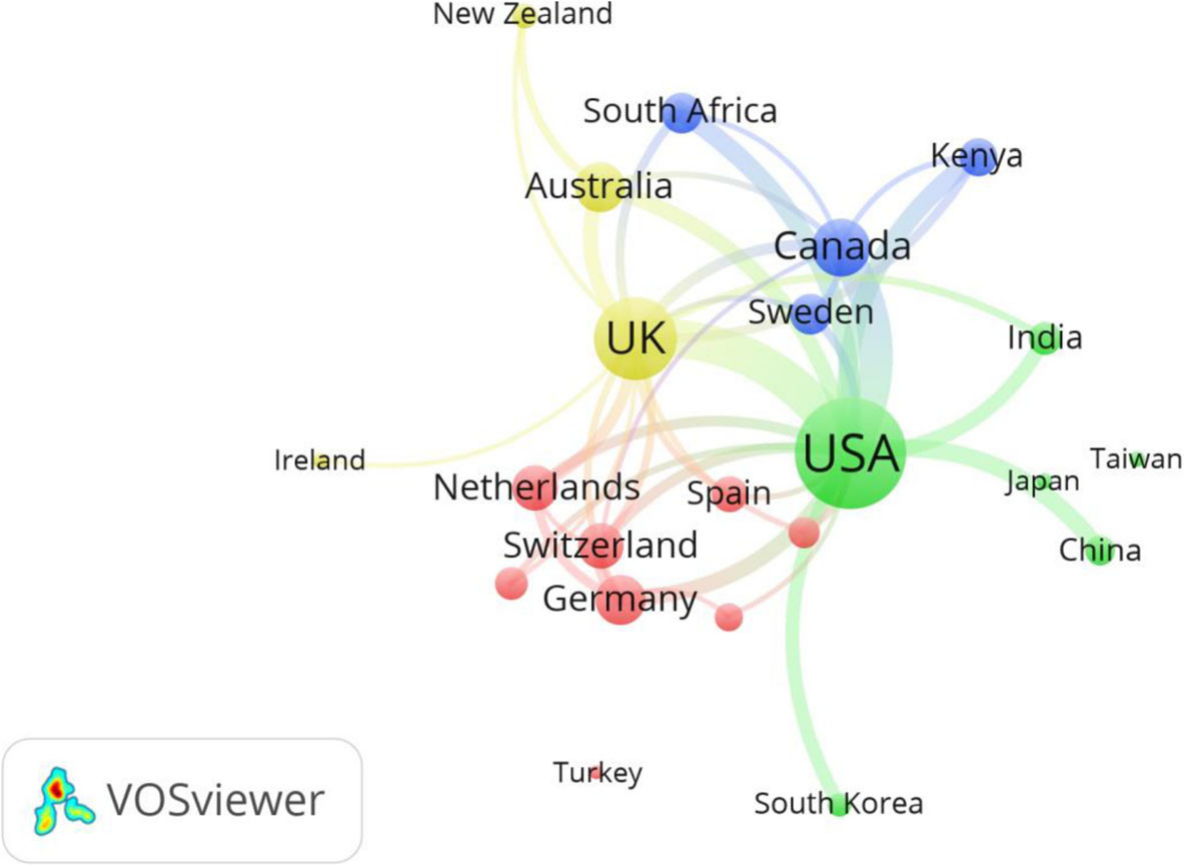
Network visualization map of international collaboration among countries with a minimum productivity of 50 documents. The thickness of connecting line between any two countries indicates strength of collaboration. For example, the link strength (collaboration) between USA and UK was 71 and it represents a thick *line*. On the other hand, the line between USA and India had a link strength of 28. Countries with similar color form one cluster. For example, countries with *red* color such as Germany and Netherlands existed in one cluster and had the highest percentage of collaboration within this cluster. India, Japan, Taiwan, and South Korea were clustered in *green* since the bulk of their collaboration is with the USA, so they are grouped with USA

Visualization of collaboration among countries with minimum productivity of 50 documents was shown in Fig. 4. The map showed 22 countries distributed in four different clusters; each with a different color. The strongest collaboration was among the following pairs of countries: USA-UK (link strength = 71); USA-Canada (link strength = 62); USA-India (link strength = 28); USA-South Africa (link strength = 36); USA-China (link strength = 33); UK-Australia (link strength = 28).

Institutions/organizations involved in m-health publications and had a minimum of 30 publications in the field were presented in Table 7. Most active institutions/organizations in the field were in the USA. *VA Medical Center* in USA was the most productive institution/organization in publishing articles in m-Health and ranked first with a total of 85 documents.

**Table 7:** Institutions and organizations with a minimum productivity of 30 documents in m-Health (2006–2016)

Rank	Institution/Organization	Frequency	% (*N* = 5465)	Country
1	VA Medical Center	85	1.6	USA
2	University of Washington, Seattle	64	1.2	USA
3	The University of Sydney	60	1.1	Australia
4	University of California, San Francisco	59	1.1	USA
5	Karolinska Institutet	57	1.0	Sweden
6	The University of British Columbia	54	1.0	Canada
7	Massachusetts General Hospital	52	1.0	USA
8	London School of Hygiene & Tropical Medicine	49	0.9	UK
9	Harvard Medical School	48	0.9	USA
9	University of Pittsburgh	48	0.9	USA
9	University of Toronto	48	0.9	Canada
9	Columbia University in the City of New York	48	0.9	USA
13	Imperial College London	44	0.8	UK
14	Johns Hopkins Bloomberg School of Public Health	43	0.8	USA
15	University of California, Los Angeles	41	0.8	USA
16	University Michigan Ann Arbor	38	0.7	USA
16	University of Oxford	38	0.7	UK
18	Duke University	36	0.7	USA
19	University of Auckland	34	0.6	New Zealand
19	University of New South Wales UNSW Australia	34	0.6	Australia
21	Johns Hopkins University	33	0.6	USA
21	The University of North Carolina at Chapel Hill	33	0.6	USA
23	David Geffen School of Medicine at UCLA	31	0.6	USA
23	Northwestern University Feinberg School of Medicine	31	0.6	USA
23	University Health Network University of Toronto	31	0.6	Canada
23	UCL (University College London)	31	0.6	UK
23	University of Queensland	31	0.6	Australia
28	Brigham and Women’s Hospital	30	0.5	USA

### Preferred journals

**Fig. 5:**
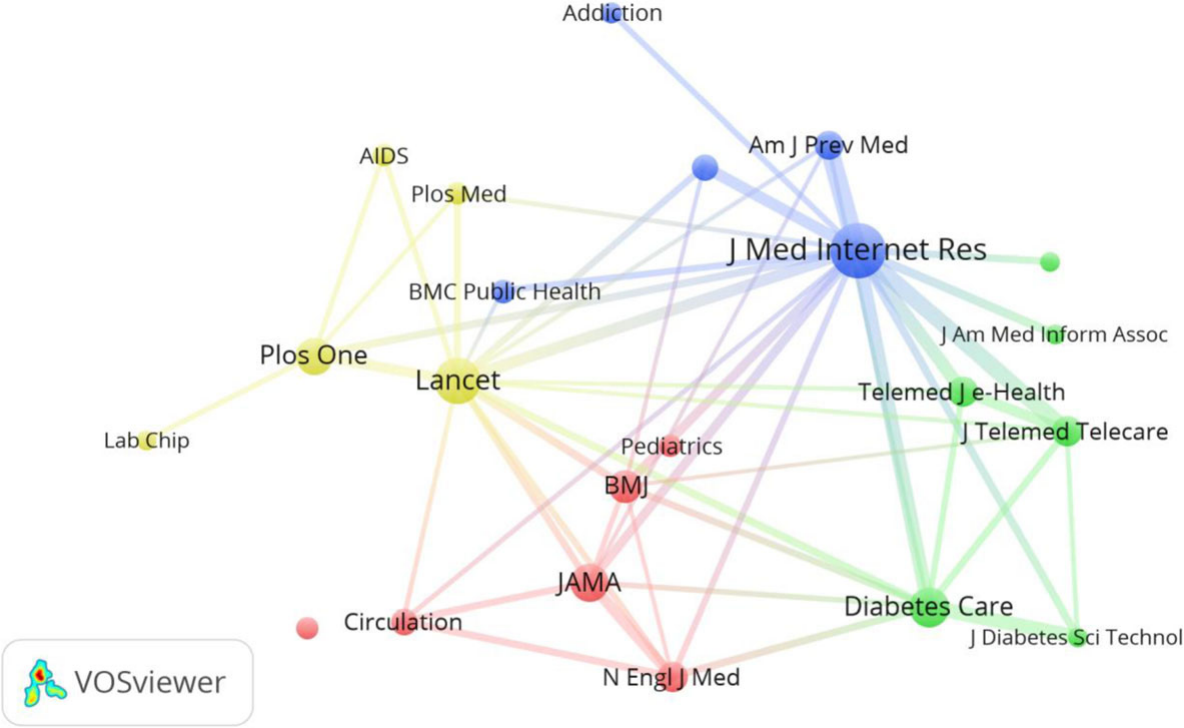
Network visualization map of journal co-citation analysis for journals which published documents in m-Health with a minimum total of 300 citations. Journal of Medical Internet Research had many connecting lines with various journals indicating that this journal is being co-cited with various journals. Journals in the same cluster with the same color are being commonly co-cited together

Journals with a minimum productivity of 20 documents were listed in Table 8. *Journal of Medical Internet Research* ranked first with 193 (3.5%) documents. Figure 5 is a network visualization map for co-citation analysis for journals with minimum citations of 300. *Journal of Medical Internet Research* received the highest number of connecting lines from other journals indicating that this journal was being co-cited with most other journals. Furthermore, the *Journal of Medical Internet Research* had the largest circle size indicative of having the highest number of citations in m-Health.

**Table 8:** Journal names with minimum productivity of 20 publications in m-Health (2006–2016)

Rank	Journal	Frequency	% *N* = 5465
1	*Journal of Medical Internet Research*	193	3.5
2	*Telemedicine and E Health*	129	2.4
3	*Plos One*	77	1.4
4	*Journal of Telemedicine and Telecare*	67	1.2
5	*Telemedicine Journal and E Health*	52	1.0
6	*Journal of Medical Systems*	50	0.9
7	*BMC Public Health*	48	0.9
8	*Annual International Conference of the IEEE Engineering in Medicine and Biology Society*	46	0.8
9	*BMC Medical Informatics And Decision Making*	40	0.7
9	*International Journal of Medical Informatics*	40	0.7
11	*Journal of Diabetes Science and Technology*	38	0.7
12	*Health Informatics Journal*	28	0.5
13	*AIDS and Behavior*	27	0.5
13	*Trials*	27	0.5
15	*Journal of Health Communication*	26	0.5
16	*BMJ Open*	25	0.5
16	*Diabetes Technology and Therapeutics*	25	0.5
18	*AMIA Annual Symposium Proceedings*	24	0.4
19	*Journal of the American Medical Informatics Association*	23	0.4
20	*Journal of the Royal Army Medical Corps*	21	0.4
20	*Sensors Switzerland*	21	0.4
22	*Lancet*	20	0.4
22	*Soins Gerontologie*	20	0.4

### Top cited documents

Top 10 cited articles in the field of m-Health were shown in Table 9. The top cited documents included seven research articles, two review articles and one editorial. The article that received the highest citation, “*Effects of a mobile phone short message service on antiretroviral treatment adherence in Kenya (WelTel Kenya1): A randomised trial*”, was published in *Lancet* in 2010 and received a total of 449 citations. The top to cited articles included two articles about antiretroviral medication adherence, one article about diabetes mellitus, one article about weight loss, one article about health behavior and the others were general articles on mobile-health. The top cited articles were published in journals in the field of internet or in general medicine.

**Table 9:** Top 10 cited articles in m-Health literature (2006–2016)

Rank	Title	Year	Journal name	Cited by	Reference	Type of document
1	*“Effects of a mobile phone short message service on antiretroviral treatment adherence in Kenya (WelTel Kenya1): A randomised trial”*	2010	*The Lancet*	449	[49]	Article
2	*“Text messaging as a tool for behavior change in disease prevention and management”*	2010	*Epidemiologic Reviews*	419	[12]	Review
3	*“Healthcare* via *cell phones: A systematic review”*	2009	*Telemedicine and e-Health*	386	[18]	Article
4	*“Mobile phone technologies improve adherence to antiretroviral treatment in a resource-limited setting: A randomized controlled trial of text message reminders”*	2011	*AIDS*	352	[59]	Article
5	*“CONSORT-EHEALTH: improving and standardizing evaluation reports of Web-based and mobile health interventions.”*	2011	*Journal of medical Internet research*	308	[60]	Editorial
6	*“How smartphones are changing the face of mobile and participatory healthcare: An overview, with example from eCAALYX”*	2011	*BioMedical Engineering Online*	299	[61]	Review
7	*“A randomized controlled trial of Sweet Talk, a text-messaging system to support young people with diabetes”*	2006	*Diabetic Medicine*	298	[62]	Article
8	*“Ecological momentary interventions: Incorporating mobile technology into psychosocial and health behaviour treatments”*	2010	*British Journal of Health Psychology*	293	[7]	Article
9	*“A text message-based intervention for weight loss: Randomized controlled trial”*	2009	*Journal of Medical Internet Research*	278	[63]	Article
10	*“Mobile phone based clinical microscopy for global health applications”*	2009	*PLoS ONE*	276	[64]	Article

## Discussion

In this study, bibliometric indicators of literature in m-Health were sought. English language remained the language of science in m-Health field with more than 93% of retrieved documents were written in English. Literature in m-Health were dominated by multi-authored publications. Data presented in our study indicated that pattern of authorship showed an increasing pattern of number of authors per document with time. Compared with other disciplines, the prevalence of multi-authored publications and collaboration index presented in our study were higher than that in the field of zoology [37], marine science [38], and information technology [35]. This might be due to the nature of the m-Health field which included two disciplines, medicine and technology, and therefore might require authors from different disciplines. Furthermore, the availability of diverse means of communication among researchers in different world regions made team research and collaboration easier and more practical which reflected positively on the prevalence of multi-authored publications seen in our study.

The USA ranked first in productivity, but contribution by European and Asian countries was also prominent. Literature in m-Health covered a wide range of health aspects including diabetes mellitus, medication adherence to anti-HIV medications, and several other aspects related to cardiovascular and chronic diseases. The *Journal of Medical Internet Research* was the most preferred journal for publishing documents in m-Health. The *VA Medical Center* in USA was the most productive institution. The volume of literature in m-health showed an exponential increase in the second half of the study, i.e., after 2009. Along with the increase in volume literature, there was an increasing trend of mean number of authors per document with time indicative of an increase in research collaboration among authors.

Mobile health applications had witnessed rapid spread in developed countries were technology and prosperous economy helped in developing healthcare services through most-up-to date technology. The advancement in technology and introduction of health application through smartphones and other mobile technologies will increase the access of people to health services and might decrease the burden on healthcare providers. For example, the new mobile health technologies allow individuals to monitor their blood pressure and heart rate and store these health data for future use. Patients with chronic diseases such as diabetes mellitus, hypertension, hemodialysis patients can use smartphones as medical records to store data regarding their diet, physical activity, life style changes, and even their day to day health changes and symptoms and present the data to healthcare providers when needed. This will help improve health system services especially in low and middle income countries where automated health system services are not well developed and numbers of healthcare providers might be limited. The power of technology in advancing health services should encourage governments, organizations, and individuals to invest in technology for better future health services. Actually, most of the emerging telecommunication technologies will be implemented directly or indirectly to health. Examples of new technologies include next-generation robotics and body-adapted wearable electronics.

The use of m-Heath in low and middle income countries might help overcome the burden of diseases and poor health services in these countries [39–41]. A systematic review study on the impact of m-Health intervention on outcome of chronic diseases reported a positive impact on chronic diseases in low and middle income countries [6]. No doubt that limited health services in many developing countries and the fast-growing mobile technology and its penetration into many low and middle income countries made the shift to m-Health in these countries to be faster than expected. An interesting example of the power of m-Health to advance health services and to reverse spread of diseases is a project called Masiluleke in South Africa which is a project that utilizes the power of communication through mobile devices to fight HIV/AIDS and tuberculosis which are major threatening public health issues for South Africa and Africa in general. The project brings powerful technology companies with non-governmental organization and the government to promote health through technology. It was not surprising that *Journal of AIDS and Behavior* was among top preferred journals for publishing documents in m-Health because technology have been invested to enhance awareness and prevention regarding AIDS. Documents published in AIDS journals focused mainly on improving adherence, risky sexual behavior, and health care for patients with AIDS [42, 43]. It was not surprising also that some of the top cited articles in the field of m-Health were in the field of medication adherence in HIV patients. Adherence to HIV medication is an important issue in medication adherence field in general and in AIDS patients in particular. A study reported that 95% or greater adherence is needed to optimize virologic outcome for patients with HIV infection [44]. Poor adherence to anti-HIV therapies is believed to be the reason for less than one third of HIV patients in developed countries with adequate viral suppression [45]. Furthermore, the importance of medication adherence in HIV patients is related to avoidance of emergence of resistance to current anti-HIV therapies [46]. The use of m-Health to optimize anti-HIV medication adherence have been notice in several African countries where healthcare services are poor and HIV is widespread [26, 42, 47–49]. Implementation of m-Health had also been applied to non-communicable diseases such as diabetes mellitus and had shown to help patients manage their glycemic control [50–52].

Our study had few limitations that are inherent to the database used and to search query developed by the authors. Such limitations were encountered in previously published bibliometric studies [53–58]. It should be emphasized that despite the fact that Scopus is one of the largest databases, there are still unindexed journals and therefore publications in these un-indexed journals might have been missed. Furthermore, no search query is 100% perfect and false positive and false negative results are always a possibility. The ranking of authors and institutions presented in our study was based on data presented by Scopus. However, in certain cases, some authors or institutions might have more than one name or different name spelling. This might create an inaccuracy in the productivity of these institutions or authors. Despite all these limitations, our study was the first to analyze bibliometric indicators of m-Health literature. Previously published bibliometric studies in the field of mobile technology [34] endorsed the findings of our study regarding growth and authorship pattern.

## Conclusion

Mobile health is an emerging and promising field that is expected to change several aspects of health services in both communicable and non-communicable diseases. Investing in m-Health should be a priority for governments, organizations and even individual patients. The emerging new mobile technologies should be tailored to help people and countries to improve national health and face major public health crisis. The data presented here will also serve for comparative future purposes to document the impact of m-Health on future research.

## Additional file

Additional file 1: Appendix 1a. List of functions available in Scopus that allows for refining of data based on various parameters. **Appendix 1b.** Source types of retrieved documents. In the current study, only journal publications were selected and analyzed. **Appendix 1c.** Types of documents showed up when retrieved data are limited to journal publications. **Appendix 1d.** Search strategy using the term “mobile health” in title-abstract-keyword. **Appendix 1e.** Search strategy using title search for words pertaining to mobile technology and health terms. **Appendix 1f.** Overall search strategy to retrieve documents in m-Health (2006–2016). Search strategy for literature in m-Health. The appendix consist of six illustrations that explain the steps and strategy implemented in Scopus database to retrieve literature in m-Health. (DOCX 599 kb)
