# Supplementary material for: Bibliometric analysis of worldwide scientific literature in mobile - health: 2006–2016
Source: BMC Med Inform Decis Mak. 2017 May 30;17:72. doi: 10.1186/s12911-017-0476-7 (PMC5450106; doi:10.1186/s12911-017-0476-7)
Supplement: Additional file 1: Appendix 1a. — List of functions available in Scopus that allows for refining of data based on various parameters. Appendix 1b. Source types of retrieved documents. In the current study, only journal publications were selected and analyzed. Appendix 1c. Types of documents showed up when retrieved data are limited to journal publications. Appendix 1d. Search strategy using the term “mobile health” in title-abstract-keyword. Appendix 1e. Search strategy using title search for words pertaining to mobile technology and health terms. Appendix 1f. Overall search strategy to retrieve documents in m-Health (2006–2016). Search strategy for literature in m-Health. The appendix consist of six illustrations that explain the steps and strategy implemented in Scopus database to retrieve literature in m-Health. (DOCX 599 kb) [file 12911_2017_476_MOESM1_ESM.docx]

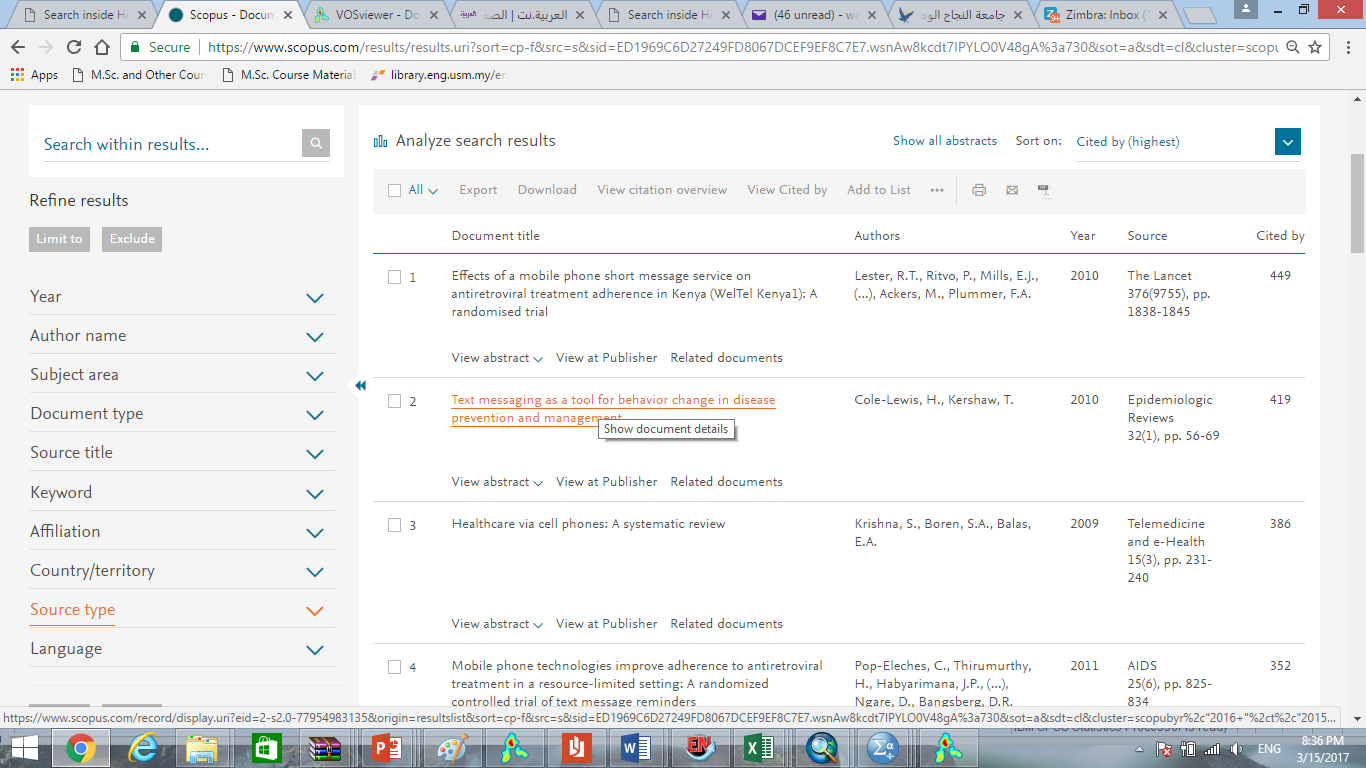


**Appendix 1a**


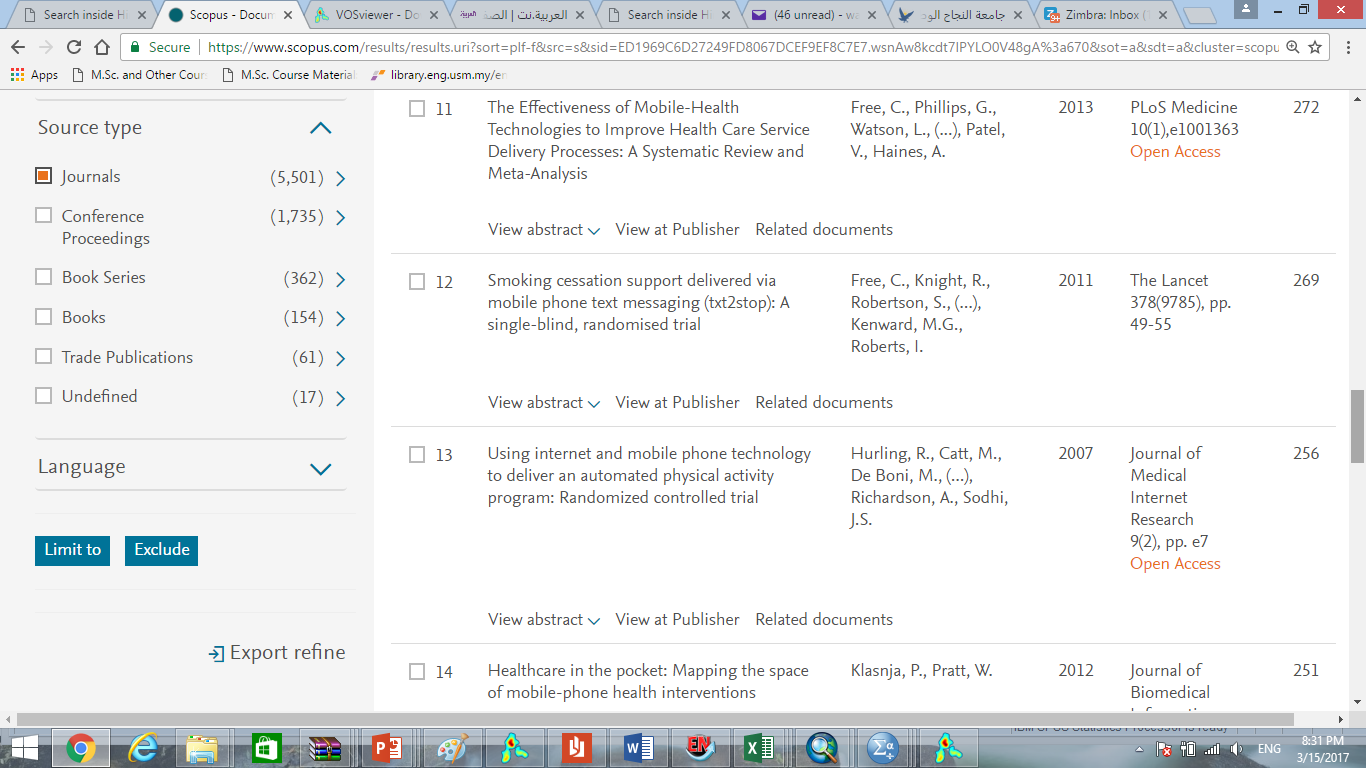


**Appendix 1b**


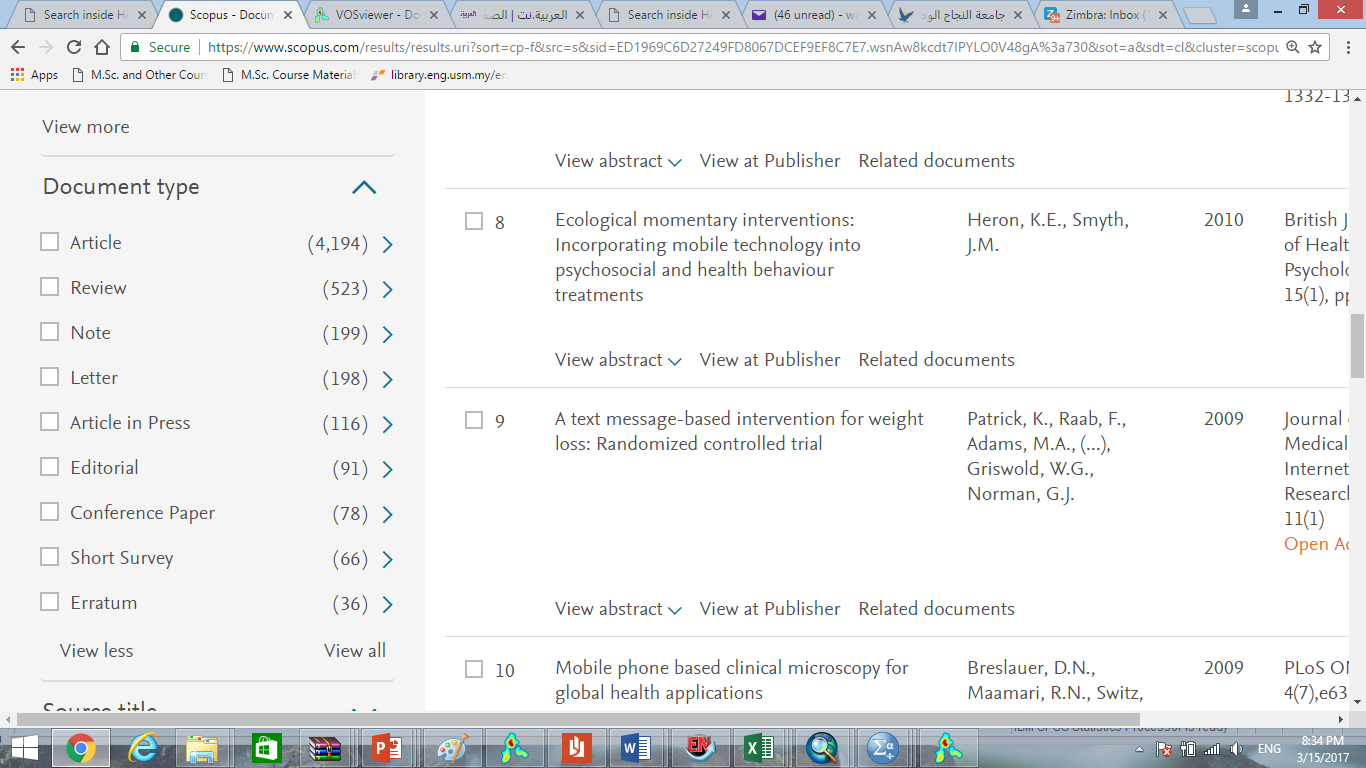


**Appendix 1c**

TITLE-ABS-KEY-AUTH ( "mobile health" ) AND ( LIMIT-TO ( SRCTYPE,"j " ) ) AND ( EXCLUDE ( DOCTYPE,"er  " ) ) AND ( LIMIT-TO ( PUBYEAR,2016 ) OR LIMIT-TO ( PUBYEAR, 2015 ) OR LIMIT-TO ( PUBYEAR, 2014 ) OR LIMIT-TO ( PUBYEAR, 2013 ) OR LIMIT-TO ( PUBYEAR, 2012 ) OR LIMIT-TO ( PUBYEAR, 2011 ) OR LIMIT-TO ( PUBYEAR, 2010 ) OR LIMIT-TO ( PUBYEAR, 2009 ) OR LIMIT-TO ( PUBYEAR, 2008 ) OR LIMIT-TO ( PUBYEAR, 2007 ) OR LIMIT-TO ( PUBYEAR, 2006 ) ) = **2452**

**Appendix 1d**

(TITLE ( "mobile technology" OR "smart phone*" OR smartphone* OR "mobile phone" OR "cell phone" OR "mobile message" OR "text messag*" OR "cellular phone" OR "mobile application*" OR "short message" OR "mobile device" OR "tablet computer" OR texting OR "personal digital assistants" OR "wireless device*" ) AND TITLE ( patient OR disease OR health* OR illness OR medication OR cardiovascular OR chronic OR surgery OR physician OR pharmacist OR pharmac* OR hospital OR nurs* OR hypertens* OR "blood pressure" OR diabet* OR "mental" OR healthcare OR medical OR medicine OR cancer OR psychiat* OR hormones OR fever OR hiv OR maternal OR pregnancy OR drug* OR dementia OR alzhem* OR neurolog* OR epilepsy OR seizure OR obesity OR "body weight" OR weight OR clinic OR "substance abuse" OR overweight OR malaria OR infecti* OR asthma OR alcohol OR clinical OR dental OR dentist OR "bipolar disorder" OR angina OR "infarction" OR schizophrenia OR ophthalmolog* OR dermatology OR "glycemic control" OR adherence OR compliance OR elderly OR therap* OR "physical activity" OR psycholg* OR smok* OR wellbeing OR emergency OR clinician OR antimicrob* OR antibiotic OR lung OR liver OR heart OR addiction OR contraception OR "family planing" OR "child health" OR headache OR migraine OR ovulat* OR fetal OR fetus OR cardiology OR "chronic disease*" OR muscul* OR oncology OR "behav* disorder*" OR prostat* OR "medical dignos*" ) ) AND ( LIMIT-TO ( SRCTYPE,"j " ) ) AND ( EXCLUDE ( DOCTYPE,"er " ) ) AND ( LIMIT-TO ( PUBYEAR,2016 ) OR LIMIT-TO ( PUBYEAR, 2015 ) OR LIMIT-TO ( PUBYEAR, 2014 ) OR LIMIT-TO ( PUBYEAR, 2013 ) OR LIMIT-TO ( PUBYEAR, 2012 ) OR LIMIT-TO ( PUBYEAR, 2011 ) OR LIMIT-TO ( PUBYEAR, 2010 ) OR LIMIT-TO ( PUBYEAR, 2009 ) OR LIMIT-TO ( PUBYEAR, 2008 ) OR LIMIT-TO ( PUBYEAR, 2007 ) OR LIMIT-TO ( PUBYEAR, 2006 ) ) = **3263**

**Appendix 1e**

Search query for “mobile health” term in title-abstract-keyword

Limit period from 2006 to 2016

Limit source type to journals

Exclude errata document

Result = 2452

Combine both queries = 5465

Search query for terms pertaining to mobile technology and health condition terms in title

Limit period from 2006 to 2016

Limit source type to journals

Exclude errata document

Result = 3263

**Appendix 1f**
